# President’s Address1Presented at the annual meeting of the Pennsylvania State Veterinary Medical Association, March 4 and 5, 1902.

**Published:** 1902-04

**Authors:** S. J. J. Harger

**Affiliations:** Philadelphia, Pa.


### PRESIDENT'S ADDRESS.[1]

By S. J. J. Harger, V.M.D.,
PHILADELPHIA, PA.

My most pleasant duty at this moment, as your presiding officer, is to welcome you, gentlemen, to the twenty-first annual meeting of this organization. For a number of years we have met at the Veterinary Department of the University of Pennsylvania; but, on account of the lack of necessary facilities, another meeting-place has been chosen. This circumstance is expected to be only temporary, and as in the past, so in the future, that insti-

1 Presented at the annual meeting of the Pennsylvania State Veterinary Medical Association, March 4 and 5, 1902.

tution will entertain the most friendly hospitality toward our organization.

Our last semi-annual meeting, as you are aware, was held in Pittsburg. Considering the attendance, the meeting may be called successful. However, I cannot disguise my thought, and I think myself justified in saying that "absence" rather than "presence" was personified, and I much regretted not to see many more familiar faces. The reason for this may be difficult to surmise. There was ample food for body and mind, and much more could have been called forth by a larger attendance. We are conscious of the benefits derived from organizations without repeating them here. We are acquainted with the advantages, not only educational but also moral and political, which this organization gives to the veterinarian of the State. I therefore hope that everyone will take an active interest in our semi-annual meetings. The veterinarian who claims that he is too busy to attend is the very one who is best able to come to these meetings. He can afford it financially, and is sufficiently strong in his community to survive the objections of his clients in leaving his practice.

During the past year veterinary science has continued its progressive tendency, the different aspects of which will be discussed to-day by the reports of the several committees. The profound sensation created by Dr. Koch on the subject of tuberculosis, and familiar to all, has died a natural death; the negative evidence was so abundant that scarcely a former shadow of itself is left. Theories come and go with such rapidity that they can be accepted only after the most careful weighing of the facts. The more sensational they are the more thought for their consideration before they should be accepted.

The bacteriologist, biologist, and microscopist still occupy the foreground; in fact, medicine might be called "microörganology." Most advances have been made in this direction, and the end is not yet in sight. We have only learned enough to know how little we know, and, on the other hand, how careful we must be before accepting conclusions. Further investigations will clear up what are now obscure points. Veterinary education is more and more shaped to answer this requirement, and with the same purpose the veterinarian should keep himself informed upon this subject. I do not mean to say that every practitioner can be an expert bacteriologist and microscopist, or that he must have a bacteriological laboratory, but that he possess sufficient knowledge to make certain practical uses to which such work can be applied.

Much can be done with the microscope, which everyone should possess, but not always does. The technique for applying it for ordinary purposes can be learned without much trouble in such cases as anæmia, leucæmia, leucocythæmia, blood parasites, anthrax, nephritis, etc., and a diagnosis is possible only with such a procedure.

The same interest is still maintained in sanitary science—meat- and milk-inspection and preventive medicine. A great deal of work has been done in this direction, and the character of this kind of work is not new to the veterinarian. The Federal Government has extended its work and increased its number of inspectors. A few individual States have done work in this direction. I believe that the veterinarians have worked and should work toward this end. They should bring the importance of this work, accompanied by facts intelligently demonstrated, to the attention of medical men and other influential persons of their community; their newspapers should be interested. This should not be done with the blare of a trumpet or by boastful audacity. These will create a reaction and excite antagonism. It requires tact, facts, and persistence.

In my previous address I spoke of the veterinarian's necessity to study the more industrial aspect of the domestic animals, judging their points for a particular utility, the special aptitude, their breeding, rearing, and feeding, and to advise those in his community who look up to him as their superior. The times have not changed.

I know that every veterinarian in the State is interested in the Veterinary Department of the University of Pennsylvania. This school was moved last fall from its old quarters to a new location. This may have given rise to the misapprehension that the school would be closed or relegated to a position secondary to that which it formerly occupied. This is not the case, and hence my remarks. It is proposed to erect on the site on which are situated at present the temporary buildings a large building that will in architecture conform with the other buildings recently erected by the University. The new school will be more conveniently located for its patrons, and its equipments will be better than those of the old one. It will combine the most approved facilities of the several schools of Europe.

I need not eulogize upon the progress of veterinary medicine. Its course is still on the ascent, and I dare say few practitioners can complain of their lot during the past year. Everyone appears to have enjoyed a reasonable degree of prosperity. That this was not local, but extended over the entire country, was exemplified by the national meeting at Atlantic City, which by old members was said to have been the most successful in the history of that Association from every point of vantage—numerically, professionally, and socially. The numerous excellent papers indicate that the veterinarian is occupying himself with scientific work; that important results are accomplished for the good of the individual and the State, and that evidently the veterinarian must receive a reasonable remuneration. The meeting was to us a striking example of encouragement.

One of the essential conditions that has always promoted the success of this Association is the harmony which has always existed among the veterinarians of Pennsylvania. This is a necessity toward success. As soon as internal strife develops in any organization its downfall can safely be predicted. Pennsylvania veterinarians have always looked at public questions in their broadest sense. They strove as one man, concentrated their energies in the same direction, and even sacrificed themselves to attain results that redound for the public good. We may need new legislation, old laws may require to be amended, appropriations for State work are needed, the influence of the veterinarian in sanitary and live-stock problems must be pushed, and our aim can only be accomplished by standing together and working as one man. The work which has been accomplished by our progeny, the State Live-stock Sanitary Board and the State Board of Veterinary Medical Examiners can be best appreciated by comparing their work with that accomplished in other States. We should be willing, without any hesitation, to give them any moral as well as financial support which they may require.

It is with extreme sorrow that I speak of the death of one of our honorary members, Rush S. Huidekoper. We all knew him, and I need not eulogize. He was perhaps the best-known veterinarian in this country. He was a man of rare ability and intellect and devoted to his profession. His energies were ceaseless in raising our profession from its dormant state, and his work will be most appreciated when he is no more. Kind of heart, he did not hesitate to do anyone a favor when it was in his power. I have been under personal obligations to him during our acquaintance, and always found in him a good friend. His decease was a great loss to veterinary science.

Finally, when I am about to leave this office, which has been intrusted to me during the past two years, let me extend my sincere thanks to this Association. I have had my shortcomings in the administration of its affairs which cannot now be remedied. I claim no credit for what has been accomplished, and perhaps much was left undone which should have been done. Every member has contributed his share of success. I wish to thank especially my co-officers for the energetic and reliable manner in which they have conducted their work, for which they deserve the highest credit. I know that my successor will meet with the same indulgence at your hands.

